# First case of COVID‐19 infused with hESC derived immunity‐ and matrix‐regulatory cells

**DOI:** 10.1111/cpr.12943

**Published:** 2020-10-26

**Authors:** Jun Wu, Zhongjie Hu, Liu Wang, Yuanqing Tan, Wei Hou, Zhongwen Li, Tingting Gao, Jiaqi Fan, Baojie Guo, Huaping Dai, Wei Li, Jie Hao, Ronghua Jin, Baoyang Hu

**Affiliations:** ^1^ National Stem Cell Resource Center Institute of Zoology Chinese Academy of Sciences Beijing China; ^2^ Institute for Stem Cell and Regeneration Chinese Academy of Sciences Beijing China; ^3^ State Key Laboratory of Stem Cell and Reproductive Biology Institute of Zoology Chinese Academy of Sciences Beijing China; ^4^ Beijing Youan Hospital Capital Medical University Beijing China; ^5^ University of Chinese Academy of Sciences Beijing China; ^6^ National Clinical Research Center for Respiratory Diseases Institute of Respiratory Medicine Chinese Academy of Medical Sciences Beijing China

**Keywords:** case report, cell therapy, COVID‐19, hESC, IMRCs

To the Editor,

Acute lung injury (ALI) and the inflammatory cytokine storm cause considerable amount of deaths in the COVID‐19 pandemic.^1^, ^2^ Currently, very limited therapeutic options are available for the COVID‐19‐induced ALI. In our preclinical experiments,^3^ we found that a single intravenous transfusion of immunity‐ and matrix‐regulatory cells (IMRCs), derived from fully differentiated human embryonic stem cells, could safely treat ALI by rapidly modulating the inflammation induced by pulmonary cell death.^3^ Encouraged by this result, as part of an expanded access programme, we pilot‐tested GMP‐grade IMRC transfusion as a compassionate treatment in a severely ill COVID‐19 patient who was diagnosed with ALI.

A 44‐year‐old male patient from Wuhan was admitted on 23 January 2020, presenting with a 6‐day history of fever and cough. The physical examination revealed a fever of 37.9°C, blood pressure of 120/61 mm Hg, pulse rate of 80 beats per minute, respiratory rate of 21 breaths per minute and blood oxygen saturation of 97.9%. Laboratory testing showed lymphocytopenia with a lymphocyte count of 0.65 × 10^9^ cells per litre. Nasopharyngeal swab specimen was collected and tested positive for SARS‐CoV‐2 by quantitative real‐time reverse transcriptase‐polymerase chain reaction (qRT‐PCR). He was briefly treated with Lianhua Qingwen (herbal flu drug) and the anti‐retroviral cocktail lopinavir/ritonavir (Kaletra), but showed no improvement. By January 28, the fever had risen to 39°C, and he had shortness of breath under oxygen supplementation. CT scans showing multiple ground‐glass opacities indicated the pneumonia had progressed to a severe stage (Figure 1A).

**Figure 1:**
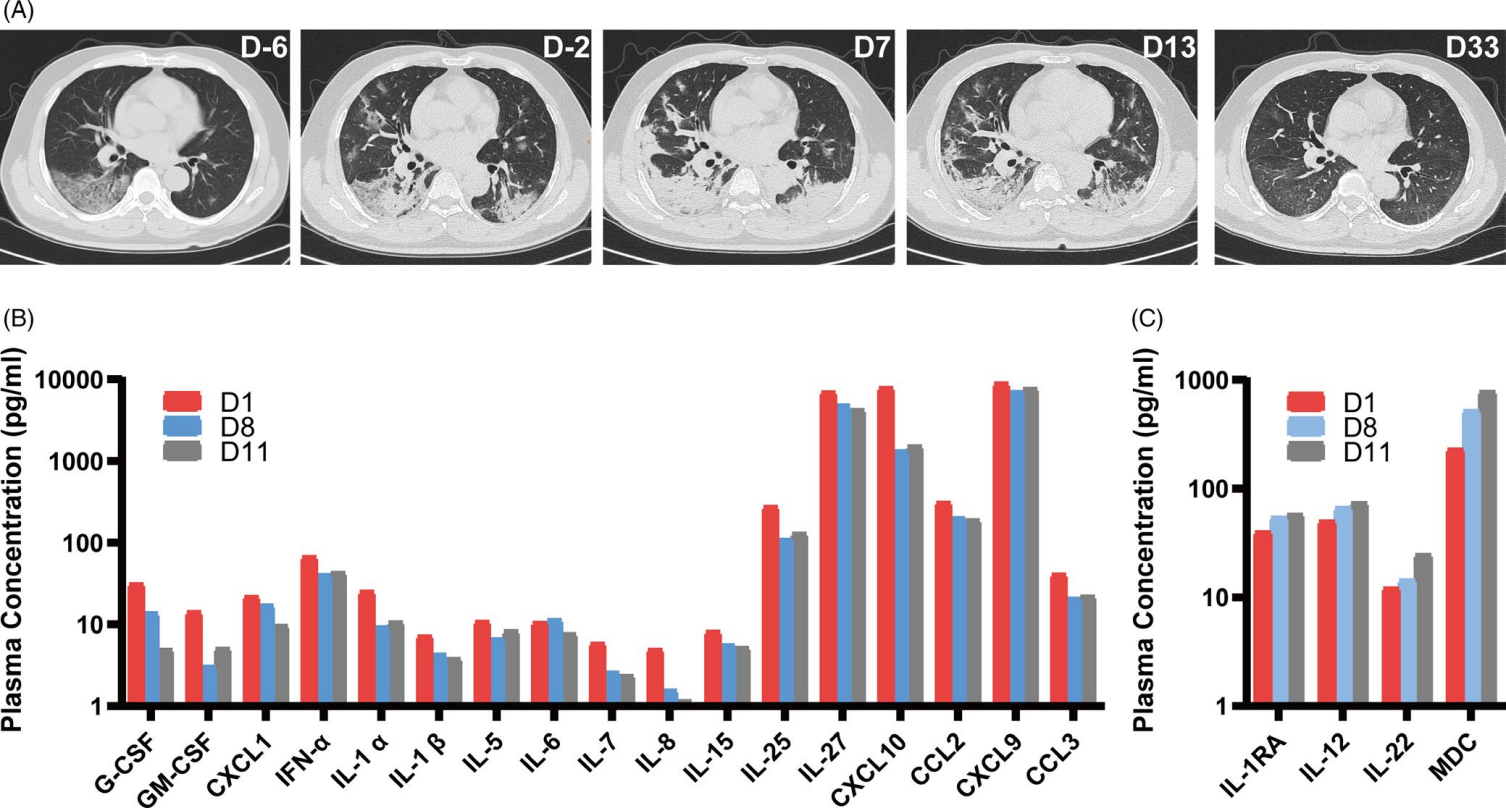
Recovery from COVID‐19‐induced acute lung injury (ALI) after immunity‐ and matrix‐regulatory cells (IMRC) infusion. Panel (A) shows a time‐series of CT scans of the patient, in terms of days before or after the first infusion. Panel (B) shows changes in representative pro‐inflammatory cytokines in the patient's plasma, between days 1‐11 after IMRC infusion. Panel (C) shows changes in the immunomodulatory cytokines in the patient's plasma, between days 1‐11 after IMRC infusion

On January 30, his resting blood oxygen saturation fell to 91%, and he was diagnosed as a severely ill patient with ALI. After the patient's consent, he was intravenously infused with 3 × 10^6^ IMRCs per kilogram body weight the same day. He was given a second infusion of IMRCs with the same dose mentioned above with interval seven days. The patient's blood pressure rose from 127/76 mm Hg to 158/106 mm Hg after the first IMRC infusion, and he was given 10 mg nifedipine orally on infusion days 1, 4, 5 and 6. The patient showed no signs of discomfort except for transient high blood pressure. By February 2, the patient no longer had shortness of breath, and his resting blood oxygen saturation rose to 95%. By February 5 (first infusion day 7), the patient's throat swab tested negative for SARS‐CoV‐2 by qRT‐PCR, which was reconfirmed on February 7. However, chest CT scans showed that the pneumonia was still in the advanced stage.

On hospital day 15 (illness day 21, first infusion day 8), the patient still had lymphocytopenia with a lymphocyte count of 0.79 × 10^9^ cells per litre. On hospital day 17 (illness day 23, first infusion day 10), the patient recovered from lymphocytopenia with a lymphocyte count of 1.02 × 10^9^ cells per litre (Table S1). On hospital day 20 (illness day 26, first infusion day 13), after showing significant recovery by CT scans, the patient was recommended for discharge. On March 2 (first infusion day 33), one month after the first IMRC infusion, follow‐up CT scans showed that the patient had completely recovered.

Cytokines in the patient's plasma were measured on first infusion day 1, first infusion day 8 (second infusion day 1) and first infusion day 11 (second infusion day 4) (Table S2). Most pro‐inflammatory cytokines showed a marked decrease, including G‐CSF, M‐CSF, GM‐CSF, IL‐1α, IL‐1β, IL‐5, IL‐6, IL‐7, IL‐8, IL‐15 IL‐17E/IL‐25, IL‐27, IP10/CXCL10, MCP‐1/CCL2, MCP‐2/CCL7, MIG/CXCL9, MIP‐1α/CCL3 and GRO‐α/CXCL1 (Figure 1B). There was also an increase in immunomodulatory cytokines, such as IL‐1RA, IL‐12, IL‐22 and MDC/CCL22 (Figure 1C).

To the best of our knowledge, this is the first time that pure hESC‐derived IMRCs have been intravenously infused into the human body. No severe adverse events were observed one month after IMRC infusion. Further work will determine whether this therapy can be replicated widely.

## CONFLICT OF INTEREST

The authors declare that there is no conflict of interest that could be perceived as prejudicing the impartiality of the research reported.

## AUTHOR CONTRIBUTIONS

BH, RJ and JH conceived the project. ZL, TG and BG, provided the testing results of IMRCs. WH, FJ, HD and WL analysed clinical case. JW, ZH, LW and Y.T designed the research and wrote the manuscript with help from all of the authors. Drs. JW, ZH, LW and YT contributed equally to this case report. B. H., JH and R. J. are the corresponding authors of this case report. All authors read and approved the final manuscript.

## ETHICAL APPROVAL

The study is approved by the Ethics Committee of Beijing Youan Hospital, Capital Medical University, Beijing, China.

## CLINICAL TRIAL REGISTRATION

ClinicalTrials.gov Identifier: NCT04331613. https://www.clinicaltrials.gov/ct2/show/NCT04331613?term=Castem&draw=2&rank=1

## Supporting information

Table S1‐S2

## Data Availability

The data that support the findings of this study are available from the corresponding author upon reasonable request.
