# Supplementary material for: First case of COVID‐19 infused with hESC derived immunity‐ and matrix‐regulatory cells
Source: Cell Prolif. 2020 Oct 26;53(12):e12943. doi: 10.1111/cpr.12943 (PMC7645847; doi:10.1111/cpr.12943)
Supplement: Supplementary file 1 — Table S1‐S2 [file CPR-53-e12943-s001.docx]

**Supplementary Appendix**

This appendix has been provided by the authors to give readers additional information on their work.

**Contents**

**Supplementary Table 1** Clinical Laboratory Results

**Supplementary Table 2** Cytokine and chemokine proteins detected in the COVID-19 patient’s plasma

**SUPPLEMENTARY TABLE 1** Clinical Laboratory Results

| **Measure** | **Reference** | **Illness day 7** | **Illness day 12** | **Illness day 15** | **Illness day 18** | **Illness day 21** | **Illness day 23** | **Illness day 46** |
| --- | --- | --- | --- | --- | --- | --- | --- | --- |
|  | **Range** | **Haspital day 1** | **Haspital day 6** | **Haspital day 9** | **Haspital day 12** | **Haspital day 15** | **Haspital day 17** | **⎯** |
| White-cell count (×10^9 per liter) | 3.50-9.50 | 3.83 | 5.69 | 6.33 | 5.20 | 6.75 | 6.69 | 7.11 |
| Neutrophil count (×10^9 per liter) | 1.80-6.30 | 2.90 | 4.44 | 4.75 | 3.83 | 5.21 | 5.00 | 5.10 |
| Lymphocyte count (×10^9 per liter) | 1.10-3.20 | 0.65 | 0.78 | 0.79 | 0.77 | 0.79 | 1.02 | 1.42 |
| Alanine aminotransferase (U/L) | 9.00-50.00 | 80.00 | 48.00 | 75.00 | 67.00 | 43.00 | 42.00 | 47.00 |
| Aspartate aminotransferase (U/L) | 15.00-40.00 | 78.00 | 50.00 | 68.00 | 47.00 | 31.00 | 36.00 | 37.00 |
| Albumin (g/L) | 40.00-55.00 | 33.60 | 26.90 | 28.00↓ | 29.60 | 34.60 | 35.60 | 42.60 |
| Estimated glomerular filtration rate (mL/min/L) | ＞90.00 | 104.40 | 112.50 | 115.50 | 113.20 | 108.50 | 110.40 | 116.20 |
| Creatinine (μmol/L) | 57.00-97.00 | 78.00 | 65.00 | 61.00 | 64.00 | 71.00 | 68.00 | 60.00 |
| Potassium (mmol/L) | 3.50-5.30 | 3.61 | 3.32 | 3.34 | 4.04 | 4.76 | 4.39 | 3.78 |
| Sodium (mmol/L) | 137.00-147.00 | 134.50 | 133.7 | 135.80 | 136.20 | 135.50 | 134.70 | 139.60 |
| Chloride (mmol/L) | 99.00-110.00 | 99.00 | 97.20 | 98.10 | 99.20 | 101.10 | 103.00 | 102.20 |
| Anion gap (mmol/L) | 10.00-14.00 | 13.80 | 6.30 | 6.20 | 7.50 | 7.00 | 6.30 | 8.60 |
| Creatine kinase (U/L) | 50.00-310.00 | 80.00 | 105.00 | 34.00 | ⎯ | ⎯ | 25.00 | 67.00 |
| Creatine kinase MB (ng/mL) | ＜3.60 | 0.09 | 0.33 | 0.00 | ⎯ | ⎯ | 0.28 | 0.17 |
| Troponin (ng/mL) | ＜0.06 | 0.01 | 0.01 | 0.01 | ⎯ | ⎯ | 0.02 | 0.02 |
| Procalcitonin (ng/mL) | ＜0.10 | 0.11 | 0.11 | ⎯ | 0.14 | 0.14 | 0.16 | ⎯ |
| C-Reactive Protein (mg/mL) | ＜3.00 | 11.80 | 80.80 | 38.90 | 50.20 | 26.40 | 19.40 | ⎯ |
| Prothrombin Time (S) | 9.90-12.80 | 12.00 | 12.80 | 12.70 | 12.30 | ⎯ | 12.50 | ⎯ |
| Prothrombin Activity (%) | 80.00-120.00 | 81.00 | 74.00 | 74.00 | 78.00 | ⎯ | 76.00 | ⎯ |
| Fibrinogen (g/L) | 2.00-4.00 | 3.58 | 4.21 | 5.22 | 5.04 | ⎯ | 5.87 | ⎯ |
| Thrombin Time (S) | 11.00-18.00 | 14.70 | 16.00 | 14.90 | 14.80 | ⎯ | 14.00 | ⎯ |

**SUPPLEMENTARY TABLE 2** Cytokine and chemokine proteins detected in the COVID-19 patient’s plasma

| **Factors** | **Unit** | **First infusion day** | | |
| --- | --- | --- | --- | --- |
|  |  | **1** | **8** | **11** |
| sCD40L | pg/ml | 2593.00 | 1453.00 | 2689.00 |
| Eotaxin | pg/ml | 162.30 | 179.40 | 162.04 |
| FLT-3L | pg/ml | 12.35 | 13.33 | 17.40 |
| Fractalkine/CX3CL1 | pg/ml | 31.72 | <31.10↓ | <31.10↓ |
| G-CSF | pg/ml | 31.60 | 13.88 | <4.94↓ |
| GM-CSF | pg/ml | 14.31 | <3.08↓ | 5.11 |
| GRO-α/CXCL1 | pg/ml | 22.18 | 17.31 | 9.73 |
| IFN-α2 | pg/ml | 67.61 | 41.04 | 43.38 |
| IFN-γ | pg/ml | 15.79 | 12.13 | 18.07 |
| IL-1α | pg/ml | 25.48 | 9.35 | 10.74 |
| IL-1β | pg/ml | 7.27 | 4.33 | 3.85 |
| IL-1RA | pg/ml | 40.50 | 54.65 | 58.52 |
| IL-2 | pg/ml | <0.53↓ | <0.53↓ | <0.53↓ |
| IL-3 | pg/ml | 4.09 | 3.10 | 3.64 |
| IL-4 | pg/ml | 0.68 | <0.50↓ | <0.50↓ |
| IL-5 | pg/ml | 10.91 | 6.69 | 8.34 |
| IL-6 | pg/ml | 10.58 | 11.53 | 7.73 |
| IL-7 | pg/ml | 5.89 | 2.61 | 2.39 |
| IL-8 | pg/ml | 4.95 | 1.57 | 1.17 |
| IL-9 | pg/ml | 11.83 | 7.95 | 7.37 |
| IL-10 | pg/ml | 18.65 | 5.53 | 11.42 |
| IL-12(p40) | pg/ml | 50.56 | 66.88 | 74.44 |
| IL-12(p70) | pg/ml | 6.66 | 4.82 | 5.68 |
| IL-13 | pg/ml | 9.51 | 7.33 | 15.91 |
| IL-15 | pg/ml | 8.24 | 5.65 | 5.22 |
| IL-17A | pg/ml | <0.94↓ | <0.94↓ | <0.94↓ |
| IL-17E/IL-25 | pg/ml | 279.22 | 109.78 | 130.88 |
| IL-17F | pg/ml | <7.41↓ | <7.41↓ | <7.41↓ |
| IL-18 | pg/ml | 38.78 | 48.59 | 50.74 |
| IL-22 | pg/ml | <12.17↓ | 14.48 | 24.91 |
| IL-27 | pg/ml | 7048.00 | 4838.00 | 4272.00 |
| IP-10/CXCL10 | pg/ml | 7993.00 | 1343.00 | 1534.00 |
| MCP-1/CCL2 | pg/ml | 312.60 | 203.13 | 191.10 |
| MCP-3/CCL7 | pg/ml | 82.57 | 43.67 | 55.26 |
| M-CSF | pg/ml | 154.29 | 59.76 | 59.76 |
| MDC/CCL22 | pg/ml | 232.26 | 524.96 | 787.60 |
| MIG/CXCL9 | pg/ml | 9032.00 | 7091.00 | 7811.00 |
| MIP-1α/CCL3 | pg/ml | 41.68 | 20.95 | 22.29 |
| MIP-1β/CCL4 | pg/ml | 97.51 | 116.48 | 126.69 |
| PDGF-AA | pg/ml | 4829.00 | 3872.00 | 4567.00 |
| PDGF-AB/BB | pg/ml | 19873.00 | 16144.00 | 18466.00 |
| RANTES | pg/ml | 3457.00 | 3843.00 | 3468.00 |
| TGF-α | pg/ml | 4.53 | 3.02 | 3.17 |
| TNF-α | pg/ml | 54.80 | 49.25 | 58.92 |
| TNF-β | pg/ml | 3.43 | 5.96 | 6.42 |
| VEGF-A | pg/ml | 364.04 | 292.99 | 308.30 |
